# *cis*-Dichloridobis(triisopropoxy­phosphine)­platinum(II)

**DOI:** 10.1107/S1600536809042226

**Published:** 2009-10-17

**Authors:** Alexandra M. Z. Slawin, Paul G. Waddell, J. Derek Woollins

**Affiliations:** a Department of Chemistry, University of St Andrews, St Andrews, KY16 9ST, Scotland

## Abstract

The title compound, [PtCl_2_(C_9_H_21_O_3_P)_2_], was obtained from a solution of PtCl_2_(COD) (COD = 1,5-cyclooctadiene) and triisopropyl­phosphite in dichloro­methane. The complex features a Pt(II) atom coordinated by two Cl and two P atoms, yielding a slightly distorted *cis* square-planar geometry.

## Related literature

For the structure of *cis*-bis­(trimethoxy­phosphite)dichlorido­platinum, see: Bao *et al.* (1987 ▶), for *cis*-dichlorido­bis(di­meth­oxy­phenyl­phosphino)platinum(II), see: Slawin *et al.* (2007*a* ▶); for dichloridobis(methoxy­diphenyl­phosphino)platinum(II), see: Slawin *et al.* (2007*b* ▶) and for *cis*-bis­(trimethoxy­phos­phite)dichlorido­palladium(II), see Slawin *et al.* (2009 ▶).
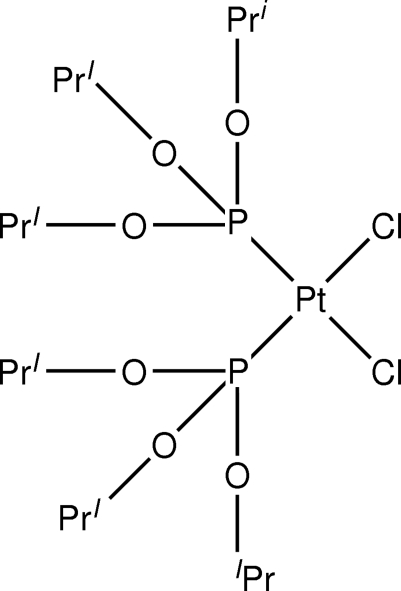


## Experimental

### 

#### Crystal data

[PtCl_2_(C_9_H_21_O_3_P)_2_]
*M* *_r_* = 682.47
Monoclinic, 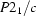

*a* = 10.8962 (4) Å
*b* = 18.9114 (8) Å
*c* = 14.2754 (6) Å
β = 104.7461 (10)°
*V* = 2844.7 (2) Å^3^
*Z* = 4
Mo *K*α radiation
μ = 5.24 mm^−1^
*T* = 125 K
0.22 × 0.22 × 0.13 mm

#### Data collection

Rigaku SCXmini diffractometer
Absorption correction: multi-scan (*ABSCOR*; Higashi, 1995 ▶) *T*_min_ = 0.356, *T*_max_ = 0.506
24157 measured reflections
4995 independent reflections
4473 reflections with *I* > 2σ(*I*)
*R*_int_ = 0.040

#### Refinement

*R*[*F* ^2^ > 2σ(*F* ^2^)] = 0.024
*wR*(*F* ^2^) = 0.038
*S* = 1.11
4995 reflections
275 parameters
H-atom parameters constrained
Δρ_max_ = 0.57 e Å^−3^
Δρ_min_ = −0.49 e Å^−3^

### 

Data collection: *SCXmini* (Rigaku, 2006 ▶); cell refinement: *PROCESS-AUTO* (Rigaku, 1998 ▶); data reduction: *PROCESS-AUTO*; program(s) used to solve structure: *SHELXS97* (Sheldrick, 2008 ▶); program(s) used to refine structure: *SHELXL97* (Sheldrick, 2008 ▶); molecular graphics: *CrystalStructure* (Rigaku, 2006 ▶); software used to prepare material for publication: *CrystalStructure*.

## Supplementary Material

Crystal structure: contains datablocks General, I. DOI: 10.1107/S1600536809042226/fi2086sup1.cif

Structure factors: contains datablocks I. DOI: 10.1107/S1600536809042226/fi2086Isup2.hkl

Additional supplementary materials:  crystallographic information; 3D view; checkCIF report

## Figures and Tables

**Table:** 

Pt1—Cl1	2.3548 (7)
Pt1—Cl2	2.3547 (9)
Pt1—P1	2.2176 (7)
Pt1—P2	2.2117 (8)

**Table:** 

Cl1—Pt1—Cl2	87.18 (2)
Cl1—Pt1—P1	171.35 (2)
Cl1—Pt1—P2	90.80 (2)
Cl2—Pt1—P1	85.34 (2)
Cl2—Pt1—P2	175.09 (3)
P1—Pt1—P2	96.99 (3)

**Fig. 1.**
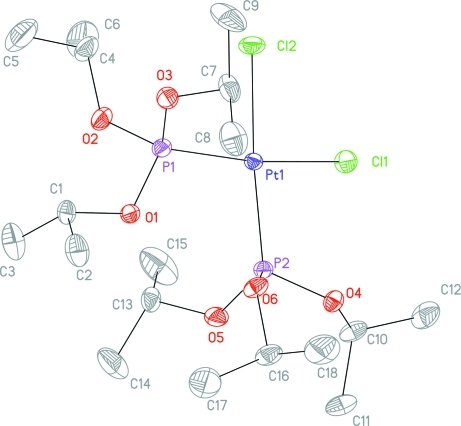
The structure of (1) with displacement ellipsoids drawn at the 50% probability level, hydrogen atoms omitted for clarity.

## supplementary crystallographic information

### Comment

The title complex can be compared to similar platinum dichloride complexes containing trimethoxy phosphite; [PtCl_2_(P(OMe)_3_)_2_] (Bao *et al.*, 1987). When compared to the structure of [PtCl_2_(P(OMe)Ph_2_)_2_] and [PtCl_2_(P(OMe)_2_Ph)_2_] (Slawin *et al.*, 2007*a*, 2007*b*) we note that the title compound has marginally shorter coordination bond lengths and significantly reduced Cl—Pt—Cl and P—Pt—P angles. Cl(1)—Pt(1)—Cl(2) 87.18?(2) , P(1)—Pt(1)—P(2) 96.99?(3) °

### Experimental

0.5 g (1.34 mmol) of PtCl_2_(COD) was dissolved in the minimum volume of dichloromethane in a round-bottomed flask. To this 0.52 mL (2.67 mmol) of triisopropylphosphite was added. The solution was stirred for 0.5 h at room temperature. The product was precipitated *via* slow diffusion of hexane and the product was filtered off and dried under vacuum, [PtCl_2_(P(O^i^Pr)_3_)_2_](0.82 mmol,*ca* 63%). ^31^P-{^1^H}NMR: δ 61.7 p.p.m.. *J*{Pt—P} 5812 Hz.

### Refinement

All H atoms were included in calculated positions (C—H distances are 0.98 Å for methyl H atoms, 1.00 Å for methylene H atoms) and were refined as riding atoms with *U*_iso_(H) = 1.2 *U*_eq_(parent atom, methylene H atoms) or *U*_iso_(H) = 1.5 *U*_eq_(parent atom, methyl H atoms). The highest peak in the difference map is 1.23 Å from atom Pt1.

### Crystal data

**Table:**

[PtCl_2_(C_9_H_21_O_3_P)_2_]	*F*(000) = 1360.00
*M**_r_* = 682.47	*D*_x_ = 1.593 Mg m^−^^3^
Monoclinic, *P*2_1_/*c*	Mo *K*α radiation, λ = 0.71075 Å
Hall symbol: -P 2ybc	Cell parameters from 27042 reflections
*a* = 10.8962 (4) Å	θ = 3.0–27.5°
*b* = 18.9114 (8) Å	µ = 5.24 mm^−^^1^
*c* = 14.2754 (6) Å	*T* = 125 K
β = 104.7461 (10)°	Chip, colourless
*V* = 2844.7 (2) Å^3^	0.22 × 0.22 × 0.13 mm
*Z* = 4	

### Data collection

**Table:**

Rigaku SCXmini diffractometer	4995 independent reflections
Radiation source: fine-focus sealed tube	4473 reflections with *F*^2^ > 2σ(*F*^2^)
Detector resolution: 6.85 pixels mm^-1^	*R*_int_ = 0.040
ω scans	θ_max_ = 25.0°, θ_min_ = 2.1°
Absorption correction: multi-scan (*ABSCOR*; Higashi, 1995)	*h* = −12→12
*T*_min_ = 0.356, *T*_max_ = 0.506	*k* = −22→22
24157 measured reflections	*l* = −16→16

### Refinement

**Table:**

Refinement on *F*^2^	H-atom parameters constrained
*R*[*F*^2^ > 2σ(*F*^2^)] = 0.024	*w* = 1/[σ^2^(*F*_o_^2^) + (0.0077*P*)^2^ + 2.3925*P*] where *P* = (*F*_o_^2^ + 2*F*_c_^2^)/3
*wR*(*F*^2^) = 0.038	(Δ/σ)_max_ = 0.001
*S* = 1.11	Δρ_max_ = 0.57 e Å^−^^3^
4995 reflections	Δρ_min_ = −0.49 e Å^−^^3^
275 parameters	

### Special details

**Table:**

Geometry. ENTER SPECIAL DETAILS OF THE MOLECULAR GEOMETRY
Refinement. Refinement was performed using all reflections. The weighted *R*-factor (*wR*) and goodness of fit (*S*) are based on *F*^2^. *R*-factor (gt) are based on *F*. The threshold expression of *F*^2^ > 2.0 σ(*F*^2^) is used only for calculating *R*-factor (gt).

### Fractional atomic coordinates and isotropic or equivalent isotropic displacement parameters (Å^2^)

**Table:**

	*x*	*y*	*z*	*U*_iso_*/*U*_eq_	
Pt(1)	0.111011 (11)	0.242171 (6)	0.268469 (9)	0.01376 (4)	
Cl(1)	0.15473 (8)	0.36409 (4)	0.26788 (6)	0.02227 (18)	
Cl(2)	−0.09964 (8)	0.27341 (4)	0.26386 (7)	0.0280 (2)	
P(1)	0.04057 (8)	0.13168 (4)	0.25627 (6)	0.01632 (18)	
P(2)	0.31368 (8)	0.21699 (4)	0.28526 (6)	0.01501 (17)	
O(1)	0.14345 (19)	0.07376 (10)	0.25384 (16)	0.0175 (5)	
O(2)	−0.0612 (2)	0.11456 (11)	0.15873 (16)	0.0234 (5)	
O(3)	−0.0298 (2)	0.10821 (11)	0.33539 (17)	0.0221 (5)	
O(4)	0.4129 (2)	0.27767 (10)	0.32742 (15)	0.0180 (5)	
O(5)	0.3566 (2)	0.19463 (12)	0.19174 (17)	0.0256 (5)	
O(6)	0.3592 (2)	0.15798 (11)	0.36474 (16)	0.0200 (5)	
C(1)	0.1080 (3)	−0.00243 (16)	0.2408 (2)	0.0216 (8)	
C(2)	0.1982 (3)	−0.04269 (17)	0.3196 (2)	0.0326 (9)	
C(3)	0.1142 (4)	−0.02438 (18)	0.1407 (2)	0.0384 (10)	
C(4)	−0.1988 (3)	0.12485 (18)	0.1375 (2)	0.0311 (9)	
C(5)	−0.2575 (3)	0.0519 (2)	0.1211 (3)	0.0587 (14)	
C(6)	−0.2359 (4)	0.1716 (2)	0.0491 (3)	0.0513 (12)	
C(7)	0.0033 (3)	0.13724 (18)	0.4343 (2)	0.0279 (8)	
C(8)	0.1093 (3)	0.0943 (2)	0.4968 (2)	0.0382 (10)	
C(9)	−0.1176 (3)	0.1367 (2)	0.4670 (3)	0.0449 (11)	
C(10)	0.4641 (3)	0.32852 (17)	0.2685 (2)	0.0228 (8)	
C(11)	0.5885 (3)	0.29936 (19)	0.2570 (2)	0.0315 (9)	
C(12)	0.4805 (3)	0.39815 (17)	0.3221 (2)	0.0321 (9)	
C(13)	0.2760 (3)	0.16206 (17)	0.1040 (2)	0.0225 (8)	
C(14)	0.3629 (4)	0.1203 (2)	0.0582 (3)	0.0442 (11)	
C(15)	0.2054 (4)	0.2179 (2)	0.0382 (2)	0.0534 (12)	
C(16)	0.4941 (3)	0.13800 (17)	0.3989 (2)	0.0253 (8)	
C(17)	0.5077 (3)	0.0630 (2)	0.3695 (3)	0.0533 (13)	
C(18)	0.5323 (3)	0.1501 (2)	0.5057 (2)	0.0466 (11)	
H(1)	0.0195	−0.0089	0.2473	0.026*	
H(2)	0.2847	−0.0377	0.3120	0.039*	
H(3)	0.1744	−0.0928	0.3155	0.039*	
H(4)	0.1945	−0.0239	0.3828	0.039*	
H(5)	0.0548	0.0043	0.0924	0.046*	
H(6)	0.0912	−0.0744	0.1306	0.046*	
H(7)	0.2005	−0.0173	0.1338	0.046*	
H(8)	−0.2217	0.1481	0.1937	0.037*	
H(9)	−0.2317	0.0288	0.0677	0.070*	
H(10)	−0.3501	0.0560	0.1049	0.070*	
H(11)	−0.2286	0.0237	0.1802	0.070*	
H(12)	−0.1912	0.2169	0.0627	0.062*	
H(13)	−0.3277	0.1799	0.0326	0.062*	
H(14)	−0.2128	0.1484	−0.0054	0.062*	
H(15)	0.0324	0.1872	0.4322	0.034*	
H(16)	0.0835	0.0446	0.4955	0.046*	
H(17)	0.1283	0.1119	0.5636	0.046*	
H(18)	0.1850	0.0984	0.4721	0.046*	
H(19)	−0.1809	0.1667	0.4239	0.054*	
H(20)	−0.1007	0.1547	0.5334	0.054*	
H(21)	−0.1500	0.0882	0.4648	0.054*	
H(22)	0.4031	0.3342	0.2035	0.027*	
H(23)	0.6462	0.2918	0.3210	0.038*	
H(24)	0.6265	0.3331	0.2204	0.038*	
H(25)	0.5733	0.2543	0.2219	0.038*	
H(26)	0.3979	0.4146	0.3288	0.038*	
H(27)	0.5160	0.4332	0.2857	0.038*	
H(28)	0.5383	0.3918	0.3864	0.038*	
H(29)	0.2141	0.1293	0.1225	0.027*	
H(30)	0.4276	0.1518	0.0444	0.053*	
H(31)	0.3135	0.0991	−0.0023	0.053*	
H(32)	0.4041	0.0829	0.1026	0.053*	
H(33)	0.1510	0.2441	0.0712	0.064*	
H(34)	0.1530	0.1959	−0.0205	0.064*	
H(35)	0.2660	0.2505	0.0208	0.064*	
H(36)	0.5458	0.1693	0.3674	0.030*	
H(37)	0.4570	0.0321	0.4000	0.064*	
H(38)	0.5971	0.0490	0.3902	0.064*	
H(39)	0.4778	0.0589	0.2989	0.064*	
H(40)	0.5205	0.2001	0.5192	0.056*	
H(41)	0.6217	0.1373	0.5311	0.056*	
H(42)	0.4797	0.1209	0.5367	0.056*	

### Atomic displacement parameters (Å^2^)

**Table:**

	*U*^11^	*U*^22^	*U*^33^	*U*^12^	*U*^13^	*U*^23^
Pt(1)	0.01280 (7)	0.01280 (7)	0.01598 (7)	0.00090 (6)	0.00422 (5)	0.00039 (6)
Cl(1)	0.0217 (4)	0.0136 (4)	0.0319 (5)	−0.0003 (3)	0.0075 (4)	0.0018 (3)
Cl(2)	0.0155 (4)	0.0193 (4)	0.0505 (6)	0.0030 (3)	0.0111 (4)	−0.0011 (3)
P(1)	0.0143 (4)	0.0152 (4)	0.0197 (5)	−0.0008 (3)	0.0047 (3)	0.0000 (3)
P(2)	0.0131 (4)	0.0171 (4)	0.0152 (4)	0.0002 (3)	0.0043 (3)	−0.0004 (3)
O(1)	0.0145 (12)	0.0139 (11)	0.0237 (13)	−0.0001 (8)	0.0044 (10)	−0.0013 (9)
O(2)	0.0168 (12)	0.0244 (12)	0.0254 (14)	−0.0020 (10)	−0.0012 (11)	−0.0037 (10)
O(3)	0.0226 (13)	0.0195 (12)	0.0279 (14)	−0.0059 (9)	0.0131 (11)	−0.0031 (10)
O(4)	0.0178 (12)	0.0175 (11)	0.0187 (12)	−0.0038 (9)	0.0046 (10)	0.0006 (9)
O(5)	0.0174 (13)	0.0394 (14)	0.0214 (13)	−0.0001 (10)	0.0076 (11)	−0.0091 (10)
O(6)	0.0140 (12)	0.0178 (11)	0.0258 (14)	0.0004 (9)	0.0004 (10)	0.0073 (9)
C(1)	0.0244 (19)	0.0115 (16)	0.030 (2)	−0.0046 (14)	0.0082 (17)	−0.0032 (13)
C(2)	0.046 (2)	0.0159 (18)	0.034 (2)	0.0017 (17)	0.008 (2)	0.0006 (16)
C(3)	0.059 (2)	0.023 (2)	0.031 (2)	−0.0006 (18)	0.008 (2)	−0.0051 (16)
C(4)	0.017 (2)	0.0241 (19)	0.044 (2)	0.0016 (15)	−0.0071 (18)	−0.0037 (17)
C(5)	0.027 (2)	0.035 (2)	0.103 (4)	−0.0081 (19)	−0.005 (2)	−0.005 (2)
C(6)	0.046 (2)	0.041 (2)	0.052 (3)	0.010 (2)	−0.015 (2)	0.002 (2)
C(7)	0.040 (2)	0.0227 (19)	0.026 (2)	−0.0053 (16)	0.0177 (19)	−0.0063 (15)
C(8)	0.053 (2)	0.036 (2)	0.026 (2)	−0.0003 (19)	0.013 (2)	−0.0005 (17)
C(9)	0.057 (3)	0.041 (2)	0.052 (3)	0.007 (2)	0.042 (2)	0.002 (2)
C(10)	0.0162 (19)	0.0271 (19)	0.026 (2)	−0.0051 (14)	0.0073 (16)	0.0091 (15)
C(11)	0.017 (2)	0.041 (2)	0.038 (2)	−0.0048 (16)	0.0108 (18)	0.0048 (18)
C(12)	0.027 (2)	0.024 (2)	0.045 (2)	−0.0072 (16)	0.0079 (19)	0.0070 (17)
C(13)	0.027 (2)	0.0254 (19)	0.0166 (19)	−0.0076 (15)	0.0085 (16)	−0.0053 (14)
C(14)	0.054 (2)	0.047 (2)	0.036 (2)	0.014 (2)	0.019 (2)	−0.011 (2)
C(15)	0.074 (3)	0.065 (3)	0.021 (2)	0.030 (2)	0.012 (2)	0.004 (2)
C(16)	0.0150 (19)	0.0242 (19)	0.034 (2)	0.0035 (14)	0.0006 (17)	0.0058 (15)
C(17)	0.034 (2)	0.039 (2)	0.076 (3)	0.014 (2)	−0.004 (2)	−0.009 (2)
C(18)	0.030 (2)	0.060 (2)	0.041 (2)	0.012 (2)	−0.006 (2)	0.003 (2)

### Geometric parameters (Å, °)

**Table:**

Pt(1)—Cl(1)	2.3548 (7)	C(4)—H(8)	1.000
Pt(1)—Cl(2)	2.3547 (9)	C(5)—H(9)	0.980
Pt(1)—P(1)	2.2176 (7)	C(5)—H(10)	0.980
Pt(1)—P(2)	2.2117 (8)	C(5)—H(11)	0.980
P(1)—O(1)	1.574 (2)	C(6)—H(12)	0.980
P(1)—O(2)	1.577 (2)	C(6)—H(13)	0.980
P(1)—O(3)	1.582 (2)	C(6)—H(14)	0.980
P(2)—O(4)	1.587 (2)	C(7)—H(15)	1.000
P(2)—O(5)	1.580 (2)	C(8)—H(16)	0.980
P(2)—O(6)	1.579 (2)	C(8)—H(17)	0.980
O(1)—C(1)	1.491 (3)	C(8)—H(18)	0.980
O(2)—C(4)	1.466 (4)	C(9)—H(19)	0.980
O(3)—C(7)	1.472 (4)	C(9)—H(20)	0.980
O(4)—C(10)	1.477 (4)	C(9)—H(21)	0.980
O(5)—C(13)	1.470 (3)	C(10)—H(22)	1.000
O(6)—C(16)	1.475 (3)	C(11)—H(23)	0.980
C(1)—C(2)	1.500 (4)	C(11)—H(24)	0.980
C(1)—C(3)	1.506 (5)	C(11)—H(25)	0.980
C(4)—C(5)	1.513 (5)	C(12)—H(26)	0.980
C(4)—C(6)	1.509 (5)	C(12)—H(27)	0.980
C(7)—C(8)	1.505 (4)	C(12)—H(28)	0.980
C(7)—C(9)	1.505 (6)	C(13)—H(29)	1.000
C(10)—C(11)	1.510 (5)	C(14)—H(30)	0.980
C(10)—C(12)	1.510 (4)	C(14)—H(31)	0.980
C(13)—C(14)	1.504 (5)	C(14)—H(32)	0.980
C(13)—C(15)	1.490 (5)	C(15)—H(33)	0.980
C(16)—C(17)	1.497 (5)	C(15)—H(34)	0.980
C(16)—C(18)	1.491 (5)	C(15)—H(35)	0.980
C(1)—H(1)	1.000	C(16)—H(36)	1.000
C(2)—H(2)	0.980	C(17)—H(37)	0.980
C(2)—H(3)	0.980	C(17)—H(38)	0.980
C(2)—H(4)	0.980	C(17)—H(39)	0.980
C(3)—H(5)	0.980	C(18)—H(40)	0.980
C(3)—H(6)	0.980	C(18)—H(41)	0.980
C(3)—H(7)	0.980	C(18)—H(42)	0.980
			
Cl(2)···C(11)^i^	3.410 (3)	H(19)···H(12)^ii^	2.981
C(7)···C(15)^ii^	3.591 (5)	H(19)···H(23)^i^	3.144
C(11)···Cl(2)^iii^	3.410 (3)	H(19)···H(36)^i^	2.880
C(15)···C(7)^iv^	3.591 (5)	H(19)···H(38)^i^	3.233
Pt(1)···H(34)^ii^	3.155	H(19)···H(41)^i^	2.992
Pt(1)···H(35)^ii^	3.571	H(20)···Cl(2)^ii^	3.557
Cl(1)···H(1)^v^	3.035	H(20)···C(2)^vi^	3.340
Cl(1)···H(3)^v^	3.574	H(20)···H(3)^vi^	2.748
Cl(1)···H(6)^v^	3.551	H(20)···H(4)^vi^	3.036
Cl(1)···H(11)^v^	3.162	H(20)···H(12)^ii^	2.693
Cl(1)···H(17)^iv^	2.893	H(20)···H(33)^ii^	3.275
Cl(1)···H(31)^ii^	3.369	H(20)···H(41)^i^	3.034
Cl(1)···H(34)^ii^	3.231	H(21)···C(2)^vi^	3.364
Cl(2)···H(3)^v^	2.807	H(21)···C(8)^vi^	3.505
Cl(2)···H(6)^v^	3.238	H(21)···H(3)^vi^	3.215
Cl(2)···H(20)^iv^	3.557	H(21)···H(4)^vi^	2.643
Cl(2)···H(23)^i^	3.099	H(21)···H(16)^vi^	2.635
Cl(2)···H(24)^i^	3.101	H(21)···H(36)^i^	3.595
Cl(2)···H(25)^i^	3.479	H(21)···H(38)^i^	2.791
P(2)···H(35)^ii^	3.584	H(21)···H(41)^i^	3.024
O(4)···H(30)^ii^	3.339	H(22)···C(18)^iv^	3.473
O(4)···H(35)^ii^	3.567	H(22)···H(17)^iv^	3.309
O(6)···H(35)^ii^	3.185	H(22)···H(40)^iv^	3.269
C(2)···H(20)^vi^	3.340	H(22)···H(42)^iv^	2.845
C(2)···H(21)^vi^	3.364	H(23)···Cl(2)^iii^	3.099
C(2)···H(24)^vii^	3.170	H(23)···C(6)^xii^	3.256
C(2)···H(41)^viii^	3.076	H(23)···H(12)^xii^	3.457
C(3)···H(5)^ix^	3.389	H(23)···H(13)^xii^	3.010
C(3)···H(9)^ix^	3.526	H(23)···H(14)^xii^	2.801
C(3)···H(14)^ix^	3.383	H(23)···H(19)^iii^	3.144
C(5)···H(26)^x^	3.186	H(24)···Cl(2)^iii^	3.101
C(5)···H(31)^ix^	3.298	H(24)···C(2)^xiii^	3.170
C(6)···H(23)^xi^	3.256	H(24)···C(18)^iv^	2.992
C(6)···H(28)^xi^	3.159	H(24)···H(2)^xiii^	2.711
C(6)···H(40)^xi^	3.542	H(24)···H(3)^xiii^	2.739
C(7)···H(33)^ii^	3.138	H(24)···H(40)^iv^	2.879
C(7)···H(34)^ii^	3.535	H(24)···H(41)^iv^	2.747
C(7)···H(35)^ii^	3.525	H(24)···H(42)^iv^	2.839
C(8)···H(16)^vi^	3.382	H(25)···Cl(2)^iii^	3.479
C(8)···H(21)^vi^	3.505	H(25)···C(18)^iv^	3.507
C(8)···H(33)^ii^	3.230	H(25)···H(8)^iii^	3.106
C(8)···H(35)^ii^	3.369	H(25)···H(13)^iii^	3.455
C(9)···H(3)^vi^	3.420	H(25)···H(40)^iv^	2.933
C(9)···H(4)^vi^	3.281	H(25)···H(41)^iv^	3.554
C(9)···H(12)^ii^	3.277	H(25)···H(42)^iv^	3.497
C(9)···H(16)^vi^	3.475	H(26)···C(5)^v^	3.186
C(9)···H(38)^i^	3.447	H(26)···C(14)^ii^	3.456
C(9)···H(41)^i^	3.199	H(26)···H(9)^v^	3.390
C(10)···H(42)^iv^	3.489	H(26)···H(10)^v^	2.928
C(11)···H(40)^iv^	3.285	H(26)···H(11)^v^	2.748
C(11)···H(41)^iv^	3.543	H(26)···H(30)^ii^	3.263
C(11)···H(42)^iv^	3.416	H(26)···H(31)^ii^	2.802
C(12)···H(10)^v^	3.574	H(27)···C(17)^xiii^	3.273
C(12)···H(13)^xii^	3.519	H(27)···H(2)^xiii^	2.916
C(12)···H(30)^ii^	3.497	H(27)···H(7)^xiii^	3.151
C(12)···H(31)^ii^	3.453	H(27)···H(10)^v^	3.541
C(12)···H(39)^xiii^	3.582	H(27)···H(32)^xiii^	3.257
C(14)···H(9)^ix^	3.453	H(27)···H(37)^xiii^	3.318
C(14)···H(10)^iii^	3.262	H(27)···H(38)^xiii^	3.323
C(14)···H(26)^iv^	3.456	H(27)···H(39)^xiii^	2.675
C(14)···H(28)^iv^	3.479	H(28)···C(6)^xii^	3.159
C(15)···H(15)^iv^	2.759	H(28)···C(14)^ii^	3.479
C(15)···H(17)^iv^	3.369	H(28)···H(7)^xiii^	3.399
C(15)···H(18)^iv^	3.591	H(28)···H(9)^xii^	3.454
C(16)···H(19)^iii^	3.510	H(28)···H(10)^xii^	3.201
C(17)···H(27)^vii^	3.273	H(28)···H(13)^xii^	2.603
C(18)···H(2)^viii^	3.554	H(28)···H(14)^xii^	2.862
C(18)···H(13)^xii^	3.538	H(28)···H(30)^ii^	2.934
C(18)···H(22)^ii^	3.473	H(28)···H(31)^ii^	3.245
C(18)···H(24)^ii^	2.992	H(30)···O(4)^iv^	3.339
C(18)···H(25)^ii^	3.507	H(30)···C(12)^iv^	3.497
C(18)···H(35)^ii^	3.510	H(30)···H(10)^iii^	2.971
H(1)···Cl(1)^x^	3.035	H(30)···H(13)^iii^	2.766
H(2)···C(18)^viii^	3.554	H(30)···H(26)^iv^	3.263
H(2)···H(24)^vii^	2.711	H(30)···H(28)^iv^	2.934
H(2)···H(27)^vii^	2.916	H(30)···H(40)^iv^	3.031
H(2)···H(41)^viii^	2.905	H(31)···Cl(1)^iv^	3.369
H(2)···H(42)^viii^	3.302	H(31)···C(5)^ix^	3.298
H(3)···Cl(1)^x^	3.574	H(31)···C(12)^iv^	3.453
H(3)···Cl(2)^x^	2.807	H(31)···H(9)^ix^	2.663
H(3)···C(9)^vi^	3.420	H(31)···H(10)^ix^	3.348
H(3)···H(20)^vi^	2.748	H(31)···H(11)^ix^	3.390
H(3)···H(21)^vi^	3.215	H(31)···H(26)^iv^	2.802
H(3)···H(24)^vii^	2.739	H(31)···H(28)^iv^	3.245
H(3)···H(41)^viii^	2.821	H(32)···H(9)^ix^	3.403
H(4)···C(9)^vi^	3.281	H(32)···H(10)^iii^	2.718
H(4)···H(20)^vi^	3.036	H(32)···H(27)^vii^	3.257
H(4)···H(21)^vi^	2.643	H(33)···C(7)^iv^	3.138
H(4)···H(38)^viii^	3.484	H(33)···C(8)^iv^	3.230
H(4)···H(41)^viii^	2.978	H(33)···H(15)^iv^	2.448
H(5)···C(3)^ix^	3.389	H(33)···H(17)^iv^	2.734
H(5)···H(5)^ix^	2.610	H(33)···H(18)^iv^	3.358
H(5)···H(6)^ix^	3.442	H(33)···H(20)^iv^	3.275
H(5)···H(9)^ix^	3.402	H(34)···Pt(1)^iv^	3.155
H(6)···Cl(1)^x^	3.551	H(34)···Cl(1)^iv^	3.231
H(6)···Cl(2)^x^	3.238	H(34)···C(7)^iv^	3.535
H(6)···H(5)^ix^	3.442	H(34)···H(6)^ix^	3.563
H(6)···H(14)^ix^	2.849	H(34)···H(15)^iv^	2.572
H(6)···H(34)^ix^	3.563	H(35)···Pt(1)^iv^	3.571
H(7)···H(9)^ix^	2.989	H(35)···P(2)^iv^	3.584
H(7)···H(14)^ix^	3.107	H(35)···O(4)^iv^	3.567
H(7)···H(27)^vii^	3.151	H(35)···O(6)^iv^	3.185
H(7)···H(28)^vii^	3.399	H(35)···C(7)^iv^	3.525
H(8)···H(25)^i^	3.106	H(35)···C(8)^iv^	3.369
H(9)···C(3)^ix^	3.526	H(35)···C(18)^iv^	3.510
H(9)···C(14)^ix^	3.453	H(35)···H(15)^iv^	2.797
H(9)···H(5)^ix^	3.402	H(35)···H(17)^iv^	3.139
H(9)···H(7)^ix^	2.989	H(35)···H(18)^iv^	3.018
H(9)···H(26)^x^	3.390	H(35)···H(40)^iv^	2.932
H(9)···H(28)^xi^	3.454	H(35)···H(42)^iv^	3.335
H(9)···H(31)^ix^	2.663	H(36)···H(19)^iii^	2.880
H(9)···H(32)^ix^	3.403	H(36)···H(21)^iii^	3.595
H(10)···C(12)^x^	3.574	H(37)···H(27)^vii^	3.318
H(10)···C(14)^i^	3.262	H(37)···H(37)^viii^	3.023
H(10)···H(26)^x^	2.928	H(37)···H(38)^viii^	3.544
H(10)···H(27)^x^	3.541	H(37)···H(41)^viii^	3.520
H(10)···H(28)^xi^	3.201	H(37)···H(42)^viii^	3.056
H(10)···H(30)^i^	2.971	H(38)···C(9)^iii^	3.447
H(10)···H(31)^ix^	3.348	H(38)···H(4)^viii^	3.484
H(10)···H(32)^i^	2.718	H(38)···H(19)^iii^	3.233
H(11)···Cl(1)^x^	3.162	H(38)···H(21)^iii^	2.791
H(11)···H(26)^x^	2.748	H(38)···H(27)^vii^	3.323
H(11)···H(31)^ix^	3.390	H(38)···H(37)^viii^	3.544
H(12)···C(9)^iv^	3.277	H(38)···H(42)^viii^	3.542
H(12)···H(19)^iv^	2.981	H(39)···C(12)^vii^	3.582
H(12)···H(20)^iv^	2.693	H(39)···H(27)^vii^	2.675
H(12)···H(23)^xi^	3.457	H(40)···C(6)^xii^	3.542
H(12)···H(40)^xi^	3.425	H(40)···C(11)^ii^	3.285
H(12)···H(41)^xi^	3.390	H(40)···H(12)^xii^	3.425
H(13)···C(12)^xi^	3.519	H(40)···H(13)^xii^	2.787
H(13)···C(18)^xi^	3.538	H(40)···H(22)^ii^	3.269
H(13)···H(23)^xi^	3.010	H(40)···H(24)^ii^	2.879
H(13)···H(25)^i^	3.455	H(40)···H(25)^ii^	2.933
H(13)···H(28)^xi^	2.603	H(40)···H(30)^ii^	3.031
H(13)···H(30)^i^	2.766	H(40)···H(35)^ii^	2.932
H(13)···H(40)^xi^	2.787	H(41)···C(2)^viii^	3.076
H(13)···H(41)^xi^	3.501	H(41)···C(9)^iii^	3.199
H(14)···C(3)^ix^	3.383	H(41)···C(11)^ii^	3.543
H(14)···H(6)^ix^	2.849	H(41)···H(2)^viii^	2.905
H(14)···H(7)^ix^	3.107	H(41)···H(3)^viii^	2.821
H(14)···H(23)^xi^	2.801	H(41)···H(4)^viii^	2.978
H(14)···H(28)^xi^	2.862	H(41)···H(12)^xii^	3.390
H(15)···C(15)^ii^	2.759	H(41)···H(13)^xii^	3.501
H(15)···H(33)^ii^	2.448	H(41)···H(19)^iii^	2.992
H(15)···H(34)^ii^	2.572	H(41)···H(20)^iii^	3.034
H(15)···H(35)^ii^	2.797	H(41)···H(21)^iii^	3.024
H(16)···C(8)^vi^	3.382	H(41)···H(24)^ii^	2.747
H(16)···C(9)^vi^	3.475	H(41)···H(25)^ii^	3.554
H(16)···H(16)^vi^	2.508	H(41)···H(37)^viii^	3.520
H(16)···H(21)^vi^	2.635	H(42)···C(10)^ii^	3.489
H(17)···Cl(1)^ii^	2.893	H(42)···C(11)^ii^	3.416
H(17)···C(15)^ii^	3.369	H(42)···H(2)^viii^	3.302
H(17)···H(22)^ii^	3.309	H(42)···H(22)^ii^	2.845
H(17)···H(33)^ii^	2.734	H(42)···H(24)^ii^	2.839
H(17)···H(35)^ii^	3.139	H(42)···H(25)^ii^	3.497
H(18)···C(15)^ii^	3.591	H(42)···H(35)^ii^	3.335
H(18)···H(33)^ii^	3.358	H(42)···H(37)^viii^	3.056
H(18)···H(35)^ii^	3.018	H(42)···H(38)^viii^	3.542
H(19)···C(16)^i^	3.510		
			
Cl(1)—Pt(1)—Cl(2)	87.18 (2)	C(4)—C(6)—H(12)	109.5
Cl(1)—Pt(1)—P(1)	171.35 (2)	C(4)—C(6)—H(13)	109.5
Cl(1)—Pt(1)—P(2)	90.80 (2)	C(4)—C(6)—H(14)	109.5
Cl(2)—Pt(1)—P(1)	85.34 (2)	H(12)—C(6)—H(13)	109.5
Cl(2)—Pt(1)—P(2)	175.09 (3)	H(12)—C(6)—H(14)	109.5
P(1)—Pt(1)—P(2)	96.99 (3)	H(13)—C(6)—H(14)	109.5
Pt(1)—P(1)—O(1)	115.04 (8)	O(3)—C(7)—H(15)	109.0
Pt(1)—P(1)—O(2)	114.47 (8)	C(8)—C(7)—H(15)	109.0
Pt(1)—P(1)—O(3)	115.43 (8)	C(9)—C(7)—H(15)	109.0
O(1)—P(1)—O(2)	100.50 (11)	C(7)—C(8)—H(16)	109.5
O(1)—P(1)—O(3)	107.34 (12)	C(7)—C(8)—H(17)	109.5
O(2)—P(1)—O(3)	102.31 (12)	C(7)—C(8)—H(18)	109.5
Pt(1)—P(2)—O(4)	116.80 (8)	H(16)—C(8)—H(17)	109.5
Pt(1)—P(2)—O(5)	117.90 (8)	H(16)—C(8)—H(18)	109.5
Pt(1)—P(2)—O(6)	110.65 (9)	H(17)—C(8)—H(18)	109.5
O(4)—P(2)—O(5)	101.20 (12)	C(7)—C(9)—H(19)	109.5
O(4)—P(2)—O(6)	100.33 (10)	C(7)—C(9)—H(20)	109.5
O(5)—P(2)—O(6)	108.15 (12)	C(7)—C(9)—H(21)	109.5
P(1)—O(1)—C(1)	120.80 (19)	H(19)—C(9)—H(20)	109.5
P(1)—O(2)—C(4)	127.4 (2)	H(19)—C(9)—H(21)	109.5
P(1)—O(3)—C(7)	122.4 (2)	H(20)—C(9)—H(21)	109.5
P(2)—O(4)—C(10)	125.05 (18)	O(4)—C(10)—H(22)	109.9
P(2)—O(5)—C(13)	126.2 (2)	C(11)—C(10)—H(22)	109.9
P(2)—O(6)—C(16)	121.6 (2)	C(12)—C(10)—H(22)	109.9
O(1)—C(1)—C(2)	107.3 (2)	C(10)—C(11)—H(23)	109.5
O(1)—C(1)—C(3)	108.2 (2)	C(10)—C(11)—H(24)	109.5
C(2)—C(1)—C(3)	113.1 (3)	C(10)—C(11)—H(25)	109.5
O(2)—C(4)—C(5)	106.3 (2)	H(23)—C(11)—H(24)	109.5
O(2)—C(4)—C(6)	107.0 (3)	H(23)—C(11)—H(25)	109.5
C(5)—C(4)—C(6)	113.1 (3)	H(24)—C(11)—H(25)	109.5
O(3)—C(7)—C(8)	109.3 (2)	C(10)—C(12)—H(26)	109.5
O(3)—C(7)—C(9)	105.9 (2)	C(10)—C(12)—H(27)	109.5
C(8)—C(7)—C(9)	114.4 (3)	C(10)—C(12)—H(28)	109.5
O(4)—C(10)—C(11)	107.7 (2)	H(26)—C(12)—H(27)	109.5
O(4)—C(10)—C(12)	107.0 (2)	H(26)—C(12)—H(28)	109.5
C(11)—C(10)—C(12)	112.3 (2)	H(27)—C(12)—H(28)	109.5
O(5)—C(13)—C(14)	106.6 (2)	O(5)—C(13)—H(29)	109.3
O(5)—C(13)—C(15)	109.9 (2)	C(14)—C(13)—H(29)	109.4
C(14)—C(13)—C(15)	112.3 (3)	C(15)—C(13)—H(29)	109.3
O(6)—C(16)—C(17)	108.1 (2)	C(13)—C(14)—H(30)	109.5
O(6)—C(16)—C(18)	107.1 (3)	C(13)—C(14)—H(31)	109.5
C(17)—C(16)—C(18)	114.2 (3)	C(13)—C(14)—H(32)	109.5
O(1)—C(1)—H(1)	109.4	H(30)—C(14)—H(31)	109.5
C(2)—C(1)—H(1)	109.4	H(30)—C(14)—H(32)	109.5
C(3)—C(1)—H(1)	109.4	H(31)—C(14)—H(32)	109.5
C(1)—C(2)—H(2)	109.5	C(13)—C(15)—H(33)	109.5
C(1)—C(2)—H(3)	109.5	C(13)—C(15)—H(34)	109.5
C(1)—C(2)—H(4)	109.5	C(13)—C(15)—H(35)	109.5
H(2)—C(2)—H(3)	109.5	H(33)—C(15)—H(34)	109.5
H(2)—C(2)—H(4)	109.5	H(33)—C(15)—H(35)	109.5
H(3)—C(2)—H(4)	109.5	H(34)—C(15)—H(35)	109.5
C(1)—C(3)—H(5)	109.5	O(6)—C(16)—H(36)	109.1
C(1)—C(3)—H(6)	109.5	C(17)—C(16)—H(36)	109.1
C(1)—C(3)—H(7)	109.5	C(18)—C(16)—H(36)	109.1
H(5)—C(3)—H(6)	109.5	C(16)—C(17)—H(37)	109.5
H(5)—C(3)—H(7)	109.5	C(16)—C(17)—H(38)	109.5
H(6)—C(3)—H(7)	109.5	C(16)—C(17)—H(39)	109.5
O(2)—C(4)—H(8)	110.1	H(37)—C(17)—H(38)	109.5
C(5)—C(4)—H(8)	110.1	H(37)—C(17)—H(39)	109.5
C(6)—C(4)—H(8)	110.1	H(38)—C(17)—H(39)	109.5
C(4)—C(5)—H(9)	109.5	C(16)—C(18)—H(40)	109.5
C(4)—C(5)—H(10)	109.5	C(16)—C(18)—H(41)	109.5
C(4)—C(5)—H(11)	109.5	C(16)—C(18)—H(42)	109.5
H(9)—C(5)—H(10)	109.5	H(40)—C(18)—H(41)	109.5
H(9)—C(5)—H(11)	109.5	H(40)—C(18)—H(42)	109.5
H(10)—C(5)—H(11)	109.5	H(41)—C(18)—H(42)	109.5
			
Cl(1)—Pt(1)—P(2)—O(4)	21.21 (10)	Pt(1)—P(2)—O(4)—C(10)	−93.9 (2)
Cl(1)—Pt(1)—P(2)—O(5)	−99.70 (10)	Pt(1)—P(2)—O(5)—C(13)	−25.7 (2)
Cl(1)—Pt(1)—P(2)—O(6)	135.11 (9)	Pt(1)—P(2)—O(6)—C(16)	−172.06 (19)
Cl(2)—Pt(1)—P(1)—O(1)	179.48 (10)	O(4)—P(2)—O(5)—C(13)	−154.4 (2)
Cl(2)—Pt(1)—P(1)—O(2)	63.79 (11)	O(5)—P(2)—O(4)—C(10)	35.4 (2)
Cl(2)—Pt(1)—P(1)—O(3)	−54.62 (9)	O(4)—P(2)—O(6)—C(16)	−48.1 (2)
P(1)—Pt(1)—P(2)—O(4)	−162.57 (9)	O(6)—P(2)—O(4)—C(10)	146.5 (2)
P(1)—Pt(1)—P(2)—O(5)	76.52 (10)	O(5)—P(2)—O(6)—C(16)	57.4 (2)
P(1)—Pt(1)—P(2)—O(6)	−48.67 (9)	O(6)—P(2)—O(5)—C(13)	100.7 (2)
P(2)—Pt(1)—P(1)—O(1)	−4.85 (10)	P(1)—O(1)—C(1)—C(2)	−129.3 (2)
P(2)—Pt(1)—P(1)—O(2)	−120.55 (11)	P(1)—O(1)—C(1)—C(3)	108.3 (2)
P(2)—Pt(1)—P(1)—O(3)	121.04 (9)	P(1)—O(2)—C(4)—C(5)	−115.5 (3)
Pt(1)—P(1)—O(1)—C(1)	−176.87 (19)	P(1)—O(2)—C(4)—C(6)	123.5 (2)
Pt(1)—P(1)—O(2)—C(4)	−86.4 (2)	P(1)—O(3)—C(7)—C(8)	−87.0 (3)
Pt(1)—P(1)—O(3)—C(7)	−31.2 (2)	P(1)—O(3)—C(7)—C(9)	149.3 (2)
O(1)—P(1)—O(2)—C(4)	149.7 (2)	P(2)—O(4)—C(10)—C(11)	−94.7 (2)
O(2)—P(1)—O(1)—C(1)	−53.4 (2)	P(2)—O(4)—C(10)—C(12)	144.3 (2)
O(1)—P(1)—O(3)—C(7)	98.5 (2)	P(2)—O(5)—C(13)—C(14)	−153.8 (2)
O(3)—P(1)—O(1)—C(1)	53.2 (2)	P(2)—O(5)—C(13)—C(15)	84.3 (3)
O(2)—P(1)—O(3)—C(7)	−156.2 (2)	P(2)—O(6)—C(16)—C(17)	−114.9 (3)
O(3)—P(1)—O(2)—C(4)	39.2 (2)	P(2)—O(6)—C(16)—C(18)	121.6 (2)

Symmetry codes: (i) *x*−1, *y*, *z*; (ii) *x*, −*y*+1/2, *z*+1/2; (iii) *x*+1, *y*, *z*; (iv) *x*, −*y*+1/2, *z*−1/2; (v) −*x*, *y*+1/2, −*z*+1/2; (vi) −*x*, −*y*, −*z*+1; (vii) −*x*+1, *y*−1/2, −*z*+1/2; (viii) −*x*+1, −*y*, −*z*+1; (ix) −*x*, −*y*, −*z*; (x) −*x*, *y*−1/2, −*z*+1/2; (xi) *x*−1, −*y*+1/2, *z*−1/2; (xii) *x*+1, −*y*+1/2, *z*+1/2; (xiii) −*x*+1, *y*+1/2, −*z*+1/2.
